# Use of super resolution reconstruction MRI for surgical planning in Placenta accreta spectrum disorder: Case series

**DOI:** 10.1016/j.placenta.2023.08.066

**Published:** 2023-10

**Authors:** Nada Mufti, Joanna Chappell, Patrick O'Brien, George Attilakos, Hassna Irzan, Magda Sokolska, Priya Narayanan, Trevor Gaunt, Paul D. Humphries, Premal Patel, Elspeth Whitby, Eric Jauniaux, J. Ciaran Hutchinson, Neil J. Sebire, David Atkinson, Giles Kendall, Sebastien Ourselin, Tom Vercauteren, Anna L. David, Andrew Melbourne

**Affiliations:** a Elizabeth Garret Anderson Institute for Women's Health, University College London, UK; b School of Biomedical Engineering and Imaging Sciences (BMEIS), King's College London, UK; c University College London Hospital NHS Foundation Trust, UK; d Department of Medical Physics and Biomedical Engineering, University College London Hospitals, UK; e Great Ormond Street Hospital for Children, UK; f Sheffield Teaching Hospitals NHS Foundation Trust, UK; g NIHR, Great Ormond Street Hospital, BRC, UK; h Centre for Medical Imaging, University College London, UK; i NIHR, University College London Hospitals BRC, UK

**Keywords:** Placenta accreta spectrum, Super resolution reconstruction, Magnetic resonance imaging, Bladder adherence, Placental attachment depth, Major obstetric haemorrhage

## Abstract

**Introduction:**

Comprehensive imaging using ultrasound and MRI of placenta accreta spectrum (PAS) aims to prevent catastrophic haemorrhage and maternal death. Standard MRI of the placenta is limited by between-slice motion which can be mitigated by super-resolution reconstruction (SRR) MRI. We applied SRR in suspected PAS cases to determine its ability to enhance anatomical placental assessment and predict adverse maternal outcome.

**Methods:**

Suspected PAS patients (n = 22) underwent MRI at a gestational age (weeks + days) of (32^+3^±3^+2^, range (27^+1^-38^+6^)). SRR of the placental-myometrial-bladder interface involving rigid motion correction of acquired MRI slices combined with robust outlier detection to reconstruct an isotropic high-resolution volume, was achieved in twelve. 2D MRI or SRR images alone, and paired data were assessed by four radiologists in three review rounds. All radiologists were blinded to results of the ultrasound, original MR image reports, case outcomes, and PAS diagnosis. A Random Forest Classification model was used to highlight the most predictive pathological MRI markers for major obstetric haemorrhage (MOH), bladder adherence (BA), and placental attachment depth (PAD).

**Results:**

At delivery, four patients had placenta praevia with no abnormal attachment, two were clinically diagnosed with PAS, and six had histopathological PAS confirmation. Pathological MRI markers (T2-dark intraplacental bands, and loss of retroplacental T2-hypointense line) predicting MOH were more visible using SRR imaging (accuracy 0.73), in comparison to 2D MRI or paired imaging. Bladder wall interruption, predicting BA, was only easily detected by paired imaging (accuracy 0.72). Better detection of certain pathological markers predicting PAD was found using 2D MRI (placental bulge and myometrial thinning (accuracy 0.81)), and SRR (loss of retroplacental T2-hypointense line (accuracy 0.82)).

**Discussion:**

The addition of SRR to 2D MRI potentially improved anatomical assessment of certain pathological MRI markers of abnormal placentation that predict maternal morbidity which may benefit surgical planning.

## Highlights

• Advanced MRI provides enhanced assessment in suspected placenta accreta spectrum.
• Additional pathological MRI markers are identified that predict adverse outcome.
• Prenatal prediction of adverse events is key to minimising operative complications.
• It can aid surgical planning by ensuring availability of appropriate clinical teams.
• This can improve maternal outcome and reduce cost of increased hospitalisation.

## 1 Introduction

Placenta accreta spectrum (PAS) disorders are characterised by abnormal villous tissue attachment to a scarred myometrium requiring surgical resection in cases of focal lesions and hysterectomy in large lesions [1]. Uterine remodelling after caesarean section can result in a scar defect with loss of decidua and myometrial structure allowing the placenta to develop close to the radial and arcuate arteries [1]. Failure to recognise PAS is associated with catastrophic haemorrhage and maternal death as the placenta fails to detach from the myometrium and surrounding tissues such as the bladder, and pelvic side wall [[2], [3], [4], [5], [6], [7]].

Comprehensive imaging is important to correctly manage PAS and optimise maternal outcome [8]. This is performed by interpretation of sonographic markers using 2-dimensional (2D) and doppler imaging, with MRI used an adjunct [3,9]. A standardised reporting protocol for ultrasound findings (e.g., abnormal placental lacunae, or myometrial thinning) has been published by the European Working Group on Abnormally Invasive Placenta (EW-AIP) [[10], [11], [12]]. Similarly, the Joint Society of Abdominal Radiology (SAR) and European Society of Urogenital Radiology (ESUR) have proposed standardised reporting of MRI signs such as placental heterogeneity, and T2-dark bands [8,9,13]. These markers are associated with uterine remodelling after surgery and are dependent on operator expertise [9,14]. Although both imaging modalities have similar sensitivity and specificity in identifying PAS, MRI may be a useful adjunct to suboptimal ultrasound and can improve surgical planning by offering a larger field-of-view, visualisation of the entire placenta-myometrial interface and lateral extent of abnormal placental attachment [6,15]. MRI, however, is compromised by between-slice maternal motion effects which can affect imaging quality [15,16]. To mitigate these challenges, advanced methods such as super-resolution reconstruction (SRR) are available. This uses post-acquisition image processing based on rigid motion correction of 2D slices combined with outlier detection to reconstruct an isotropic high-resolution volume [[17], [18], [19], [20], [21]]. SRR use has been found to enhance anatomical evaluation of fetal brain and neck anomalies [[17], [18], [19],^21^,^22^].

We hypothesised that superior anatomical soft tissue definition presented by the addition of SRR to standard 2D MRI may enhance prediction of more severe PAS through potential easier identification of certain MRI markers of abnormal placentation that would be associated with more adverse peri-operative events. We prospectively explored this in a case-series of women with clinical suspicion of PAS.

## 2 Methods

### 2.1 Patients

Twenty-two consecutive patients with singleton pregnancies and high clinical suspicion of PAS were prospectively recruited (2019–2022) in UCLH, a tertiary fetal medicine specialist unit attached to a PAS multidisciplinary team (MDT). All cases were initially diagnosed by a fetal medicine specialist on mid-trimester ultrasound, using the EW-AIP standardised protocol [10]. Women also underwent a PAS risk assessment which included history of previous PAS or scar ectopic pregnancies, caesarean and classical caesarean sections, surgical uterine evacuations, uterine surgery (myomectomy or endometrial ablation), and suspicion of first trimester scar pregnancy. All patients provided written consent for fetal MRI in the third trimester which was reported by a radiologist specialising in abnormal placentation. All clinical care including surgical delivery was provided by the PAS MDT team with access to cell salvage and interventional radiology. The International Federation of Gynaecology and Obstetrics (FIGO) clinical grading system and the International Society of Abnormally Invasive Placenta (IS-AIP) guideline for management of abnormally invasive placenta was used to assess PAS severity at delivery [2,3,10]. Placenta praevia was classified as non-PAS if there was complete placental separation by controlled cord traction (CCT), or simple manual removal [23]. The surgical approach was homogenous whereby all women were counselled pre-delivery for a low threshold of focal myometrial excision or caesarean hysterectomy if there was evidence of abnormal placental adherence with risk of life-threatening haemorrhage despite uterotonics and/or mechanical, surgical or radiological methods. Photographs were taken of any caesarean hysterectomy specimens with the placenta in situ. Histological analysis was performed by three expert perinatal pathologists (CH, EJ, and NS) using recently recommended histopathologic features for PAS diagnosis (thick fibrinoid deposition, distortion of the uteroplacental interface, and loss of decidua between villous tissue and myometrium) which are adapted from the FIGO clinical grading system of: grade 1 (placenta accreta), 2 (placenta increta), and 3 (placenta percreta) [1,3,12]. Clinical outcome data was classified into major obstetric haemorrhage (MOH, blood loss of >2.5 L), additional surgical interventions to control bleeding (Bakri Balloon insertion, or B-Lynch brace sutures), and bladder adherence (BA) to the lower uterine segment. Histological findings were classified as placental attachment depth (PAD). Surgical interventions to control bleeding where scored as 0 = none, 1 = performed. Bladder adherence was assessed by a senior clinical operator and was classified as adherent when it was difficult to establish a clear surgical plane between the bladder and uterus with/without abnormal neo-vascularity at the utero-vesical interface thus making it difficult to allow for non-traumatic reflection of the urinary bladder at hysterectomy. Bladder adherence was scored as either 0 = none/minimal adherence and vascularity, or 1 = moderate/severe adherence and vascularity with or without bladder injury. Minimal adherence and vascularity corresponded to the IS-AIP severity grading of 1–4, and moderate/severe adherence corresponded to the IS-AIP severity grading of 5. Placenta attachment depth was assessed based on the IS-AIP severity grading and the FIGO clinical grading system at delivery and histology respectively [3,10]. Placental attachment depth was recorded as 0 = none, 1 = superficial or deep attachment. This definition was used instead of the traditional terms (accreta, increta, and percreta) to better describe the level of villous tissue invasion into the uterine serosa and surrounding organs. This follows recent understanding that accreta placentation is not truly invasive, but that abnormal trophoblastic attachment is secondary to the defective decidual layer following scarification and distortion of the uteroplacental interface by excessive fibroid deposition due to the secondary increase in sub-placental and intervillous circulation [12,24,25]. All results were compared between those patients with placenta praevia at delivery versus those with confirmed PAS (either clinically or histologically using FIGO and IS-AIP severity grading).

### 2.2 MRI protocol and super-resolution reconstruction

All MRI examinations were performed on a 1.5 T magnet (MAGENETOM Avanto; Siemens Healthcare). Patients were placed in the left lateral decubitus position and had a moderately filled bladder. The uterus was imaged in at least 3 orthogonal planes (axial, coronal, and sagittal) relative to the placenta-myometrium interface. The protocol (specific to Siemens) consisted of T2-weighted fast acquisition spin echo sequences, typically HASTE (half-Fourier acquisition single-shot turbo spine echo), gradient echo sequences such as T2-weighted true-FISP (Fast Imaging with Steady state-free Precession), and T1-weighted inversion recovery (IR) sequences. For HASTE, the following parameters were applied: slice thickness (4 mm), spacing between slices (4 mm), repetition time (1013.8 ms), echo time (89.6 ms), flip angle (107.9°), and pixel spacing (0.8–0.8 mm). Total acquisition time did not exceed 60 min.

A home-grown SRR algorithm which is available as an open-source package (https://github.com/gift-surg/NiftyMIC) was then applied to reconstruct an isotropic 3-dimensional (3D) volume of the placenta-myometrial-bladder interface with native 2D MRI stacks [19]. At least 3 orthogonal T2-weighted image stacks, acquired in at least 3 orientations were used. A region of interest (ROI) in 1 stack around the placenta-myometrial-bladder interface was manually segmented using ITK-Snap™ (Version 3.20, 2014) and automatically propagated to the remaining stacks with rigid volume-to-volume registration [26]. Afterward, robust SRR involving iterative rigid-body motion correction and volumetric reconstruction steps was deployed, guided by the respective placenta-myometrial-bladder interface masks. Iterative 3D reconstructions were estimated from motion-corrected slices through outlier-robust SRR methods to account for image artefacts as part of the motion correction step. All images were reconstructed to an isotropic resolution of 1.5 mm. Each SRR production did not exceed 15 min.

### 2.3 SRR impact on image analysis

We conducted experiments whereby radiologists were asked to assess 2D MRI or SRR images alone, or in combination. Experiments were separated into 3 rounds. The first two rounds involved a selection of individual SRR and 2D MRI scans, which were assessed by 4 senior paediatric and gynaecologic radiologists (E.W., P.D.H., P.P., and T.G.) with specialist MRI expertise in abnormal placentation. All radiologists were blinded to results of the ultrasound, original MR image reports, case outcomes and PAS diagnosis. The selection was randomly assigned such that each radiologist had a different set of independent SRR and 2D MRI scans in the first 2 rounds. Each radiologist read all images by the end of the second round. In the third round, radiologists each examined all 2D MRI scans paired with their corresponding SRR images. The first two rounds were separated by 1 week; 2 weeks after this the third round was performed (Supplementary Information 1).

In every round, the assessment contained a set of clinical questions that had 3 themes: confidence in identifying patients with a high probability of PAS at birth, anatomical clarity, and identification of pathological MRI markers recommended by the SAR-ESUR joint consensus (Supplementary Information 2) [8]. The time taken to answer these questions was measured. PAS risk evaluation was scored on a confidence scale of 1–5 ranging from no confidence at all to completely confident. Anatomical clarity of the placenta-myometrial-bladder interface was subjectively scored 0 to 4, where 0 = structure was not seen, 1 = poor depiction, 2 = suboptimal visualisation, 3 = clear visualisation of structure but reduced tissue contrast (image-based diagnosis feasible), and 4 = excellent depiction (optimal for diagnostic purposes). Presence of various MRI markers were evaluated and included dark T2-dark intraplacental bands, placental/uterine buldge, loss of retroplacental T2-hypointense line, myometrial thinning, bladder wall interruption, focal exophytic mass, abnormal vascularisation of the placental bed, placental heterogeneity, asymmetric shape/thickening of the placenta, placental ischaemic infarction, and abnormal extraplacental vascularity. Radiologists also evaluated SRR quality, whereby 0 = substantial artefact/blur, 1 = little artefact/blur, and 2 = no artefact/blur. 2D MRI quality was not quantitatively examined, as there was no predefined marking comparable to our SRR quality scoring. Radiologists also provided a subjective preference in the third round, to indicate whether 2D MRI, SRR images, both, or neither was more superior. The NASA Task Load Index (TLX) was performed at the end of each round to quantify cognitive load of evaluating 2D MRI, and SRR images alone, and those 2 modalities paired [27]. The TLX is a subjective multidimensional tool measuring workload to assess a task and is frequently used in the context of introducing new medical technology [22,[28], [29], [30], [31]].It scores different task aspects such as effort, frustration, and performance on a scale from 0 (low) to 20 (high) (Supplementary Information 3).

### 2.4 Statistics

Previous SRR studies in fetal and maternal medicine had small numbers due recent development of the technology and interest in unique clinical applications [22]. This was therefore a pilot study investigating the clinical utility of SRR in PAS, and a sample size calculation could not be performed. We however aimed to recruit 20 patients, with successful SRR and available clinical and histopathological outcomes on at least 10. Statistical analysis was performed using Excel (Microsoft 365), SPSS Statistics for Mac version 27 (IBM Corp), and Python programming language. A Shapiro-Wilk test was used to check normality of data. A Mann-Whitney *U* test was used to compare differences between groups for demographics and clinical information with a non-normal distribution. Kruskal-Wallis 1-way analysis of variance with correction for multiple comparisons was used to assess data between imaging modalities. Results are documented as test statistic (degree of freedom) and the *P*-value. ROC curve analysis was used to display correct identification of patients at high risk of PAS at birth using all imaging methods. Results are documented as area under the curve (AUC), and 95% confidence interval. For illustration of differences among modalities, a Bland-Altmann analysis was used, with the mean difference as a bias measure and the 2.5th and 97.5th percentiles as the 95% limits of agreement. Statistical significance was set <5%. Inter-observer analysis between radiologists' assessments using each imaging modality was quantified using Pearson's correlation coefficient (r), whereby r < 0.3 indicated none or very week correlation, 0.3 < r < 0.5 weak correlation, 0.5 < r < 0.7 moderate correlation, and r > 0.7 strong correlation [32]. A Boruta algorithm based on a Random Forest Classification model (RFCM) was used to highlight the most predictive pathological MRI markers for adverse clinical outcomes (MOH, PAD, and BA) [[33], [34], [35]]. The most predictive markers were selected through repetitive statistical testing, whereby markers were compared against ‘shadow markers’ that are built based on random shuffling of the original markers. The RFCM was used instead of multinominal logistic regression analysis to prevent over fitting and reduce the influence of outliers. Pathological markers with the lowest ranking are more predictive of adverse maternal outcome. Accuracy was assigned to measure the usefulness of the pathological markers in correlation to outcomes. Model based ROC curve analysis was also used to display clinical outcome predictivity using individual, and lowest ranking MRI markers collectively, for each imaging modality.

## 3 Results

### 3.1 Patients and imaging

**Fig. 1:**
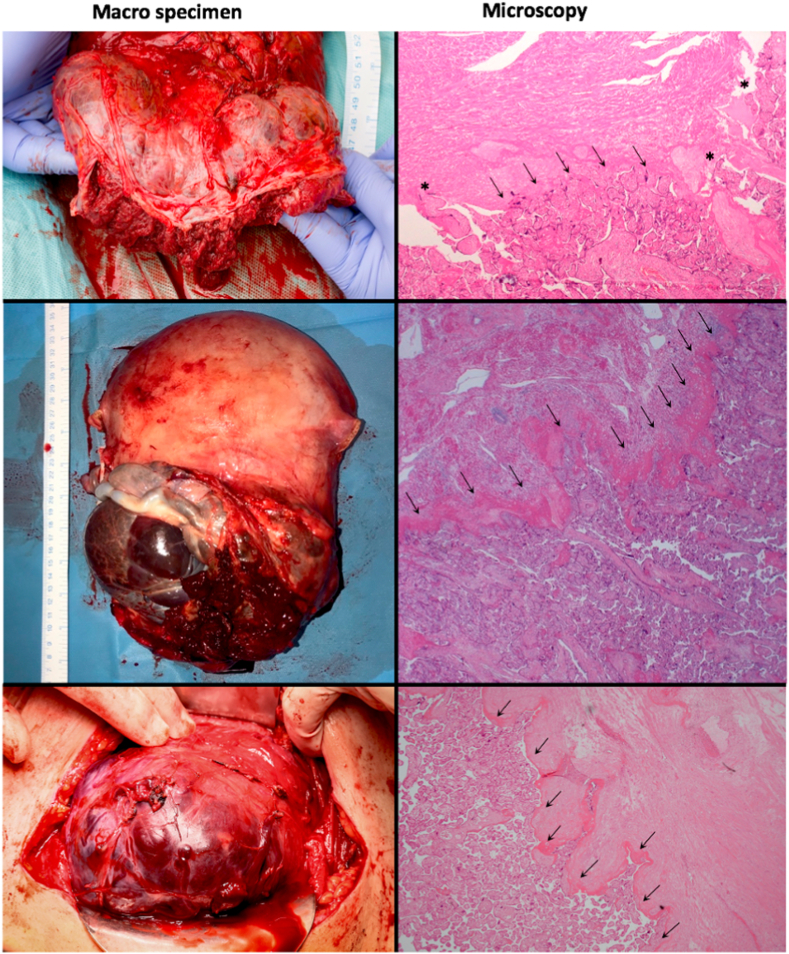
Macro specimen (left column) and Microscopy (right column) images of hysterectomy specimens for three patients diagnosed with PAS. The original magnification of the top and bottom microscopy images is x20. The original magnification of the middle microscopy image is x40. On microscopy there is fibrinoid deposition between the anchoring villi and the scarred myometrium with no intervening decidua (black arrows). Chorionic villi are deeply attached in the myometrium where they reach a serosal defect (Asterix *).

Out of twenty-two recruited patients with suspected PAS, twelve (55%) had successful MRI reconstruction and were included in the final analysis. Unsuccessful SRR was due to insufficient stacks, and/or moderate/severe motion leading to artefact and suboptimal image quality. Out of the included twelve cases, at delivery four patients had placenta praevia with no abnormal attachment, and eight were confirmed as PAS (histopathological confirmation n = 6, intraoperative clinical diagnosis n = 2). Of those six women who had histological PAS confirmation (hysterectomy n = 5, and focal myometrial excision n = 1), two met the histological criteria of FIGO grade 1, and four satisfied the criteria of FIGO grade 2 [3], (Fig. 1). The two women who had intraoperative growth signs supporting a clinical PAS diagnosis, had this performed in accordance with the IS-AIP clinical severity grading system adapted from recent FIGO guidance [10]. One of those patients had an IS-AIP clinical severity grading of 2 whereby there was no placental separation with oxytocin or CCT and manual removal of the placenta was required with the entire placenta noted to be adherent by a senior experienced clinical operator. The second patient had an intraoperative IS-AIP clinical severity grading of 3 whereby the uterus over the placenta appeared bluish in appearance with an obvious ‘placental buldge’ (10 × 10cm over the lower segment) with no signs of separation with oxytocin. Gentle CCT resulted in the ‘dimple sign’ and therefore manual removal of the placenta was required. The whole placental bed was again thought to be adherent by the same senior clinical operator.

Average gestational age (GA) (weeks + days) at MRI was (33^+3^± 4^+2^, range (27^+0^–38^+6^)) for confirmed PAS cases, and (33^+3^± 3^+2^, range (30^+0^–36^+5^)) for placenta praevia. Average GA at caesarean section was (36^+3^ ± 1^+2^, range (34^+0^ ± 38^+6^)) for PAS cases, and (36^+0^ ± 1^+1^, range (34^+0^–37^+1^)) for placenta praevia. There was no difference between groups in GA at MRI or caesarean section. The recorded blood loss (L) at delivery was significantly different between PAS (5.01 ± 4.05, 95% CI (1.63–8.4)), and the placenta praevia subgroup (1.5 ± 0.9, 95% CI (0.07–2.93)), *p* = 0.028, Supplementary Information 4. Seven out of eight patients in the PAS group had MOH >2.5 L. There were no cases of MOH in any of the four patients in the praevia subgroup.

**Fig. 2:**
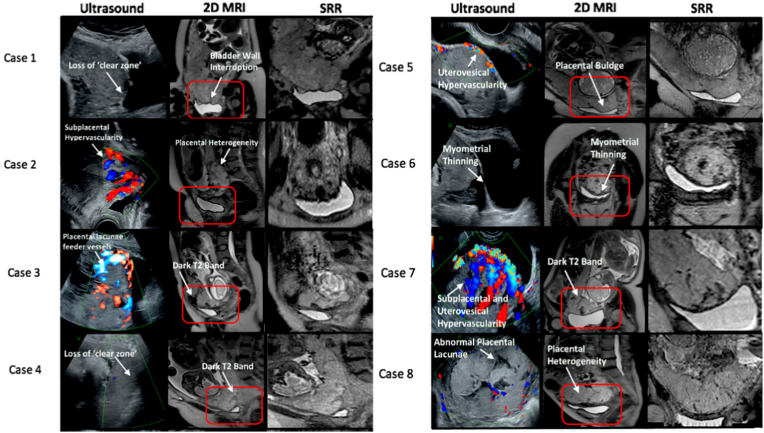
Ultrasound and magnetic resonance images of the 8 confirmed PAS cases. The first column shows ultrasound images. The box indicates the placenta-myometrial-bladder interface on the 2-dimensional (2D) MRI image (second column) that was reconstructed (third column, SRR). The arrows and labels indicate examples of pathological ultrasound and MRI markers in PAS disorder. 2D, 2-dimensional; MRI, Magnetic Resonance Imaging; SRR, Super Resolution Reconstruction.

Post MRI acquisition SRR was performed with a stack average of 4.25 ± 0.87, (range 3–6). Average slice rejection was 7.5 ± 7.04, (range 0–22), indicating the degree of between-slice maternal motion in acquired data. Fewer than 5 stacks or a large number of slice rejection is typically indicative of suboptimal SRR quality (Supplementary Information 5). The resulting ultrasound, 2D MRI, and SRR images for all PAS cases can be seen illustrated alongside in Fig. 2.

### 3.2 PAS diagnosis

ROC curve analysis showed that by using 2D MRI alone (AUC 0.94, 95% CI (0.80–1)), or SRR alone (AUC 0.92, 95% CI (0.77–1)), radiologists were more likely to correctly identify patients at high risk of PAS at birth in comparison to paired imaging (AUC 0.86, 95% CI (0.65–1)) (Supplementary Information 6).

### 3.3 Confidence regarding PAS diagnosis

**Fig. 3:**
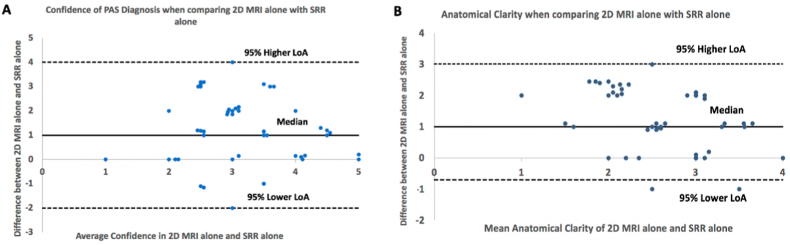
Bland-Altman plot illustrating differences in the (A): confidence of PAS diagnosis and (B): subjective anatomic clarity when using 2D MRI alone compared to using SRR imaging alone, whereby 0 on the y axis = equal confidence. 2D, 2-dimensional; MRI, Magnetic Resonance Imaging; SRR, Super Resolution Reconstruction.

Radiologists were more confident in assessing patients at high risk of PAS with 2D MRI alone in comparison with SRR images alone (test statistic (2) = 3.728, *p* < 0.001) (Fig. 3). There was no improvement in confidence between paired data and 2D MRI alone.

### 3.4 Anatomical clarity

Subjective anatomical clarity was higher when radiologists used 2D MRI alone in comparison with SRR images alone (test statistic (2) = 4.198, *p* < 0.001) (Fig. 3). There was no improvement in anatomical clarity between paired data and 2D MRI alone.

### 3.5 Predictivity of pathological MRI markers for adverse maternal outcome

**Fig. 4:**
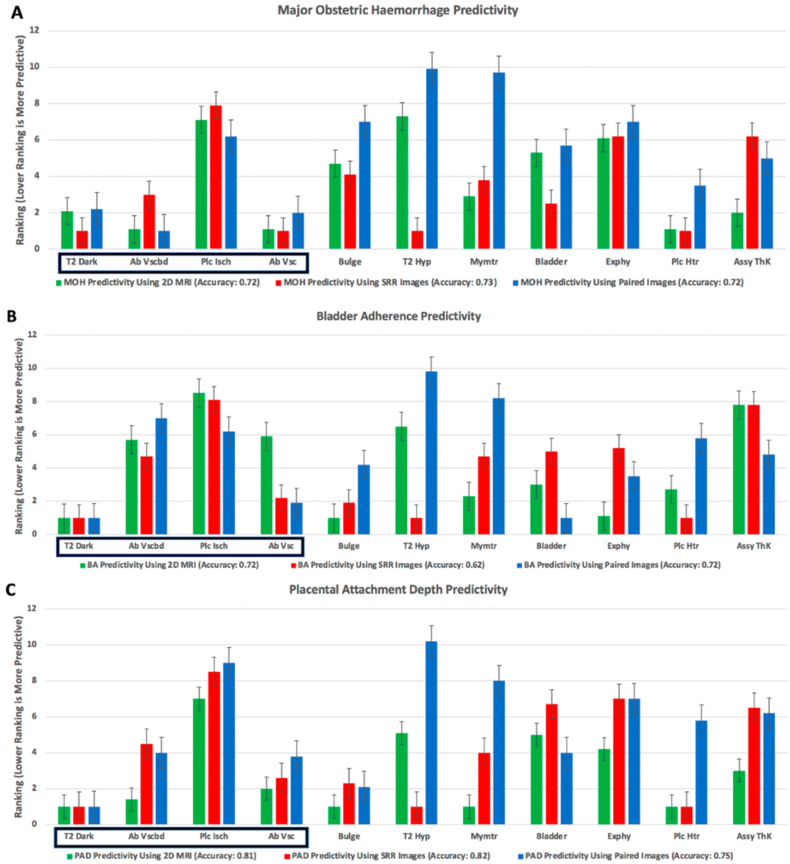
Pathological MRI marker predictivity for (A): major obstetric haemorrhage (MOH), (B): moderate/severe bladder adherence (BA), and (C): superficial/deep placental attachment depth (PAD) using 2D MRI (green), SRR (red), and Paired Imaging (blue). Markers with the lowest ranking better predicted MOH, BA, and PAD with the stated accuracy above each chart. Error bars show the variation of rank for each MRI marker. Markers are initially arranged as indicators of abnormalities of utero-placental circulation and are outlined in a black box (T2 Dark, T2-dark intraplacental bands; Ab Vscbd, Abnormal vascularisation of the placental bed; Plc Isch, Placental ischaemic infarction; Ab Vsc, Abnormal extraplacental vascularity) followed by pathological anatomical markers of placenta accreta spectrum (Bulge, Placental/uterine buldge; T2 Hyp, Loss of retroplacental T2-hypointense line; Mymtr, Myometrial thinning; Bladder, Bladder wall interruption; Exphy, Focal exophytic mass; Plc Htr, Placental heterogeneity; Assy ThK, Asymmetric shape/thickening of the placenta).

**Fig. 5:**
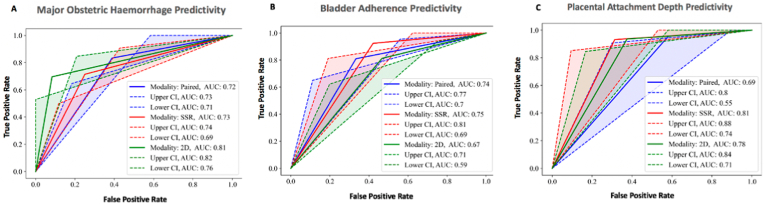
The most predictive (lowest ranking) pathological MRI marker for (A): major obstetric haemorrhage (MOH), (B): moderate/severe bladder adherence (BA), and (C): superficial/deep placental attachment depth (PAD) using 2D MRI (green), SRR (red), and Paired Imaging (blue). The most predictive markers for MOH using each imaging modality: 2D MRI (Abnormal vascularisation of the placental bed, Abnormal extraplacental vascularity, Placental heterogeneity); SRR (T2-dark intraplacental bands, Abnormal extraplacental vascularity, Loss of retroplacental T2-hypointense line, Placental heterogeneity); Paired (Abnormal vascularisation of the placental bed). The most predictive markers for BA using each imaging modality: 2D MRI (T2-dark intraplacental bands, Bulge, Focal exophytic mass); SRR (T2-dark intraplacental bands, Loss of retroplacental T2-hypointense line, Placental heterogeneity); Paired (T2-dark intraplacental bands, Bladder wall interruption). The most predictive markers for PAD using each imaging modality: 2D MRI (T2-dark intraplacental bands, Bulge, Myometrial thinning, Placental heterogeneity); SRR (T2-dark intraplacental bands, Loss of retroplacental T2-hypointense line, Placental heterogeneity); Paired (T2-dark intraplacental bands).

When radiologists used SRR alone, there were additional pathological MRI markers detected that were predictive of MOH (>2.5 L) when compared to either 2D MRI alone or paired imaging. These prognostic markers are T2-dark intraplacental bands, and loss of retroplacental T2-hypointense line (RFCM accuracy of 0.73). Markers such as placental heterogeneity, and abnormal extraplacental vascularity were also predictive of MOH using either 2D MRI alone (RFCM accuracy 0.72) or SRR alone (RFCM accuracy 0.73). Abnormal vascularisation of the placental bed was additionally predictive of MOH using 2D MRI alone, and this was maintained if paired data was used (RFCM accuracy of 0.72), (Fig. 4). Overall MOH predictivity was highest using 2D MRI alone (AUC = 0.81, 95% CI (0.76–0.82)) versus SRR alone (AUC = 0.73, 95% CI (0.69–0.74)) or paired imaging (AUC = 0.72, 95% CI (0.71–0.73)) when using the most predictive (lowest ranking) MRI markers collectively (Fig. 5).

Although SRR alone allowed for additional pathological MRI markers to be detected that were predictive of moderate/severe BA and vascularity (which may be accompanied with bladder injury) in comparison to 2D MRI alone or paired imaging, it did so with a reduced RFCM accuracy of 0.62 in comparison to 2D MRI alone or paired imaging which have a predictive RFCM accuracy of 0.72. Loss of retroplacental T2-hypointense line, and placental heterogeneity were most predictive for BA using SRR alone, whilst bladder wall interruption was most predictive using paired imaging and placental bulge was most predictive using 2D MRI alone. Detection of T2-dark intraplacental bands was most predictive for BA using any of the three imaging combinations, (Fig. 4). BA predictivity was highest using SRR alone (AUC = 0.75, 95% CI (0.69–0.81)) followed by paired imaging (AUC = 0.74, 95% CI (0.70–0.77)) versus 2D MRI alone (AUC = 0.67, 95% CI (0.59–0.71)) when using the lowest ranking MRI markers collectively (Fig. 5).

T2-dark intraplacental bands was most predictive for abnormal superficial or deep placental attachment depth (PAD) in relation to the myometrium using all imaging combinations; 2D MRI alone (0.81 RFCM accuracy), SRR alone (0.82 RFM accuracy), and paired imaging (0.72 RFCM accuracy). Pathological MRI markers such as placental heterogeneity was most predictive for PAD using either 2D MRI alone, or SRR alone. Prediction of PAD was also possible with the detection of two additional MRI markers (placental buldge and myometrial thinning) using 2D MRI alone, and one additional marker (loss of retroplacental T2-hypointense line) using SRR alone, (Fig. 4). PAD predictivity using the lowest ranking MRI markers collectively was slightly higher using SRR alone (AUC = 0.81, 95% CI (0.74–0.88)) versus 2D MRI alone (AUC = 0.78, 95% CI (0.71–0.84)). PAD predictivity using either SRR alone or 2D MRI alone was higher than paired imaging (AUC = 0.69, 95% CI (0.55–0.8)), (Fig. 5).

The least predictive pathological marker for MOH, BA, and PAD was placental ischaemic infarction using either 2D MRI alone, or SRR alone. Loss of retroplacental T2-hypointense line was least predictive for MOH and BA using 2D MRI alone, and all adverse peri-operative outcomes using paired imaging.

Predictivity for additional surgical interventions to control bleeding could not be performed due to minimal available outcome data. This is likely due to a low threshold of focal myometrial resection, or hysterectomy if there were no signs of placental detachment to minimise major obstetric haemorrhage.

ROC curve analyses for each individual MRI marker predicting adverse maternal outcome using each imaging modality are displayed in Supplementary Information 7,8, and 9.

### 3.6 Time and subjective preference

The average time (seconds) taken to answer clinical questions was not different between 2D MRI alone (358.31, 95% CI (302.82–413.8)), SRR alone (330.99, 95% CI (287.31–374.65)), or paired data (360.21, 95% CI (313.29–406.73). Radiologists preferred using 2D MRI images in comparison to SRR alone (test statistic (3) = 4.164, *p* < 0.001). There was no improvement in preference between paired data and 2D MRI alone (Supplementary Information 10).

### 3.7 Inter-observer analysis

There was a strong correlation between radiologists’ assessments using 2D MRI alone (r = 0.748, 95% CI (0.668–0.812)). SRR alone (r = 0.618, 95% CI (0.496–0.717), and paired imaging (r = 0.626, 95% CI (0.504–0.724)) showed moderate correlation.

### 3.8 NASA TLX

**Fig. 6:**
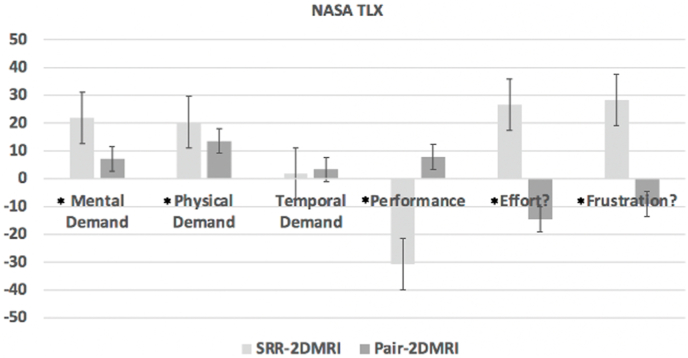
NASA TLX indicating difference between SRR alone and 2D MRI alone and between paired data and 2D MRI alone. Values are presented as mean (95% CI). 2D, 2-dimensional; MRI, magnetic resonance imaging; SRR, super-resolution reconstruction; TLX, Task Load Index.

Mental (test statistic (2) = −3.663, *p* = 0.002), physical (test statistic (2) = −3.133, *p* = 0.010), effort (test statistic (2) = −4.961, *p* < 0.001) and frustration (test statistic (2) = −7.133, *p* < 0.001) levels were higher with SRR images alone in comparison to 2D MRI alone. Performance was lower with SRR imaging alone compared to 2D MRI (test statistic (2) = −5.853, *p* < 0.001). There was no improvement of these subscales between paired data and 2D MRI alone. There was no difference between groups in temporal demand (Fig. 6).

## 4 Discussion

### 4.1 Main findings

We found that the addition of SRR to 2D MRI provided potentially improved identification of certain pathological MRI markers that are prognostic for adverse maternal outcomes in PAS, with no increase in time taken to make a clinical assessment. When using the most predictive MRI markers collectively, MOH predictivity was highest using 2D MRI alone compared to SRR alone, or paired imaging. BA predictivity on the other hand was highest using SRR alone, or paired imaging versus 2D MRI alone. There was only a slight increase in PAD predictivity by using SRR alone in comparison to 2D MRI alone. Both SRR alone and 2D MRI alone outperformed paired imaging in PAD predictivity. Radiologists were likely to correctly identify a high probability of PAS at birth using 2D MRI, or SRR alone in comparison to paired imaging. Due to higher subjective anatomical clarity, radiologists were less confident in their evaluation using SRR alone, in comparison to 2D MRI.

### 4.2 Interpretation

The prognostic ability of MRI markers for adverse peri-operative events is important given the significant maternal morbidity and mortality associated with PAS [[6], [7], [8]]. We showed that T2-dark intraplacental bands, which are predictive for MOH, was potentially easier to identify using SRR alone, compared to paired images or 2D MRI [8]. This corresponds with previous evidence showing that T2-dark bands are associated with increased intraoperative blood loss and caesarean hysterectomy [36,37]. Similarly, loss of retroplacental T2-hypointense line, which is predictive for MOH, was possibly highlighted better using SRR alone, but not paired images or 2D MRI where it was found to be least predictive. Although this marker appears thinned in regions of transient focal myometrial contractions, its presence with other signs such as T2-dark bands, abnormal extraplacental vascularity, and placental heterogeneity as detected by SRR alone may increase its prognostic sensitivity [15,38,39]. Abnormal vascularisation of the placental bed was also predictive for MOH using 2D MRI or paired data in comparison to SRR alone. However, this sign is normally seen in advanced gestations due to increase in sub-placental and myometrial vascularity and should be cautiously interpreted [15,40,41]. Nevertheless, MOH predictivity was highest using 2D MRI alone versus SRR alone or paired imaging when using the most predictive pathological markers collectively.

Bladder wall interruption was most reliant for BA predictivity and was potentially easier to identify using paired data in comparison to 2D MRI, or SRR alone. Abnormal superior traction is often seen due to abnormal placental adherence to the bladder wall [8,15]. When combined with T2-dark bands as seen in all MRI data, this sign should raise suspicion for moderate/severe BA with increased vascularity. Although SRR alone detects additional MRI markers predictive of BA such as loss of retroplacental T2-hypointense line and placental heterogeneity, this is weakly predicted. Furthermore, placental heterogeneity is frequently seen in later gestations due to fibrin and calcifications secondary to normal placental maturation [42]. Similarly, placental bulge was predictive for BA using 2D MRI alone compared to SRR or paired data. This sign however is often related to thinned overlying myometrium complying to the bulky placental contour and may not be reliable for BA predictivity [15].

When considering abnormal PAD, additional predictive markers were potentially better highlighted using 2D MRI (placental buldge, myometrial thinning) and SRR (loss of retroplacental T2-hypointense line) compared to paired imaging. However, myometrial thinning can occur in late gestation and is best used in conjunction with loss of retroplacental hypointense line which has been found to be predictive of PAS [8]. This information, along with the similarity in PAD predictivity when using the most predictive MRI markers with either SRR alone, or 2D MRI alone, as shown in our results, may promote the use of both of these imaging tools in combination which could be potentially useful for facilitation of surgical planning.

The least predictive marker for adverse maternal events was placental ischaemic infarction using all imaging modalities. This correlates with expert opinion where this finding was less suggestive for PAS due to its subjectivity and inter-reader variability [8].

This study describes how SRR, paired imaging, and 2D MRI may potentially highlight additional predictive markers for adverse maternal outcome. Although cognitive load is increased when SRR imaging is added, this may be a worthwhile compromise when considering the added prognostic ability. Reduced SRR performance in terms of confident PAS diagnosis may be explained by a natural learning curve that radiologists encounter when interpreting SRR images, given the different texture and visualisation against the original MRI. In the presence of good original image quality, and stacks in at least 3 orientations, volumetric reconstruction provides exquisite placental-myometrial-bladder interface definition. This can have immense clinical potential by facilitating surgical planning. Accurate prediction of adverse peri-operative events is key to minimising complications allowing the team to avoid unexpected surgical findings and schedule a multi-disciplinary approach. It enables careful counselling and implementation of individually tailored treatments (hysterectomy vs conservative surgery). PAS delivery is complex requiring an MDT including obstetricians, gynaecologic-oncologic surgeons, urologists, interventional radiologists, anaesthetics, haematologists, and neonatologists [8,43]. If adverse events such as MOH and PAD are predicted, delivery in a tertiary unit with surgical expertise, blood banks capable of managing massive transfusion, and readily available intensive care units is essential [44]. Similarly, blood loss can be minimised by adjusting surgical approach (e.g. fundal hysterotomy) coupled with selective arterial embolization, or intraoperative internal iliac balloon inflation [45]. If BA is predicted, urology presence is essential as bladder wall repair is challenging, often associated with massive haemorrhage, and ureteric injury [41].

### 4.3 Strengths and limitations

A main strength of our study is advanced MRI technology application in PAS and focussing on its predictive ability for adverse peri-operative events. Furthermore, we performed a prospective evaluation with histopathological confirmation. Our SRR algorithm is however limited, as the reconstruction quality is reliant on good original MRI data. The resulting small sample size may have therefore affected impacted on results. Furthermore, the underlying rigid motion reconstruction model limits the ability to compensate for complex motion outside the placental-myometrial-bladder interface. This limited ROI may have affected radiologists’ ability to identify its spatial location within the larger anatomical context. Further technical developments are needed to support full clinical translation of SRR. For instance, super-resolved images may be improved by employing a wider field-of-view to include the cervix. This has potential surgical implications if profound vascularity is found between the cervix and bladder as it involves vaginal and vesical arterial branches which are not amenable to usual endovascular control techniques [15,46]. Additionally, the current algorithm assumes unimodal imaging and incorporation of sequences such as T1-IR may provide further information. Future optimisation can also create 3D-MRI models illustrating abnormal placental adherence depth and bladder involvement which can aid surgical planning (Supplementary Information 11).

### 4.4 Conclusion

In women with suspected PAS, the addition of SRR to 2D MRI may potentially improve anatomical assessment of certain pathological MRI markers that are predictive of adverse maternal outcomes. This may benefit surgical planning by ensuring appropriate clinical teams and equipment are available. Additional technical development and validation with a larger data set is however necessary to support the reconstruction fidelity, and SRR imaging should be considered as supportive information and not a replacement of the original 2D-MRI stacks.

## Contribution to authorship

N.M. designed the study, analysed the data, wrote the first draft and corrected the final version of the manuscript. J.C. and H.I. developed and applied the Boruta algorithm based on a Random Forest Classification model (RFCM) and contributed to the writing and editing of the manuscript. G.A. performed, reported and supervised the acquisition of ultrasound imaging, and M.S. acquired all the MRI images in UCLH, London. P.N. reported all MRI images. P.O.B. counselled all patients and performed the surgeries. E.J., J.C.H., and N.S. performed all histopathological diagnoses and provided the images with advice on histopathological content in this manuscript. T.G., P.D.H., P.P., and E.W. are all trained radiologists who carried out all 3 experimental rounds. T.V., D.A., M.S., and G.K. provided technical advice on MRI and biomedical physics content in this manuscript. S.A., A.L.D. and A.M. supported and supervised this work since conception. All authors have seen and approved the final version and consent to the submission of the manuscript.

## Ethics and funding

All MRI data were analysed under the study entitled ‘‘Guided Instrumentation for Fetal Therapy and Surgery (GIFT-Surg): Fetal MRI to Improve Prenatal Diagnosis and Therapy for Fetal Abnormality’’ (Hampstead Research Ethics Committee, 15/LO/1488), funded by the 10.13039/100010269Wellcome Trust [203148/Z/16/Z; 203145Z/16/Z; WT101957] and 10.13039/501100000266Engineering and Physical Sciences Research Council (EPSRC) [NS/A000049/1; NS/A000050/1; NS/A000027/1; EP/L016478/1]. Funders had no role in the study design, collection, analysis, data interpretation, writing of the manuscript or decision to submit the article for publication. S.O. is the principal investigator on this grant, and A.L.D., T.V. and A.M. are co-investigators. N.M. is funded with support of the 10.13039/100004440Wellcome/10.13039/501100000266EPSRC centre for Interventional and Surgical Sciences (WEISS) (203145Z/16/Z). A.L.D. is supported by 10.13039/501100000272the National Institute for Health Research University College London Hospitals Biomedical Research Centre.

Women provided written informed consent for fetal MRI research. All images were transferred with Caldicott Guardian approval from University College London Hospitals (UCLH) to collaborators at partner academic institutions (University College London (UCL), King's College London (KCL)) via the secure GIFT-Cloud platform, which ensures de-identification through XNAT technology [47].

## Declaration of competing interest

None

## Abbreviations

PAS: Placenta Accreta Spectrum
SRR: Super-Resolution Reconstruction
2D: 2-Dimensional, 3D, 3-Dimensional
MRI: Magnetic Resonance Imaging
MOH: Major Obstetric Haemorrhage
BA: Bladder Adherence
PAD: Placental Attachment Depth
EW-AIP: European Working Group on Abnormally Invasive Placenta
SAR: Society of Abdominal Radiology
ESUR: European Society of Urogenital Radiology
OS: Orifice
FIGO: International Federation of Gynaecology and Obstetrics
IS-AIP: International Society of Abnormally Invasive Placenta
CCT: Controlled Cord Traction
MDT: Multidisciplinary Team
HASTE: half-Fourier acquisition single-shot turbo spin echo
True-FISP: Fast Imaging with Steady state-free Precession
IR: Inversion Recovery
ROI: Region of Interest
TLX: Task Load Index
GA: Gestational Age
AUC: Area Under the Curve
RFCM: Random Forest Classification Model
