# Supplementary material for: Use of super resolution reconstruction MRI for surgical planning in Placenta accreta spectrum disorder: Case series
Source: Placenta. 2023 Oct;142:36–45. doi: 10.1016/j.placenta.2023.08.066 (PMC10937261; doi:10.1016/j.placenta.2023.08.066)
Supplement: Multimedia component 2 [file mmc2.docx]

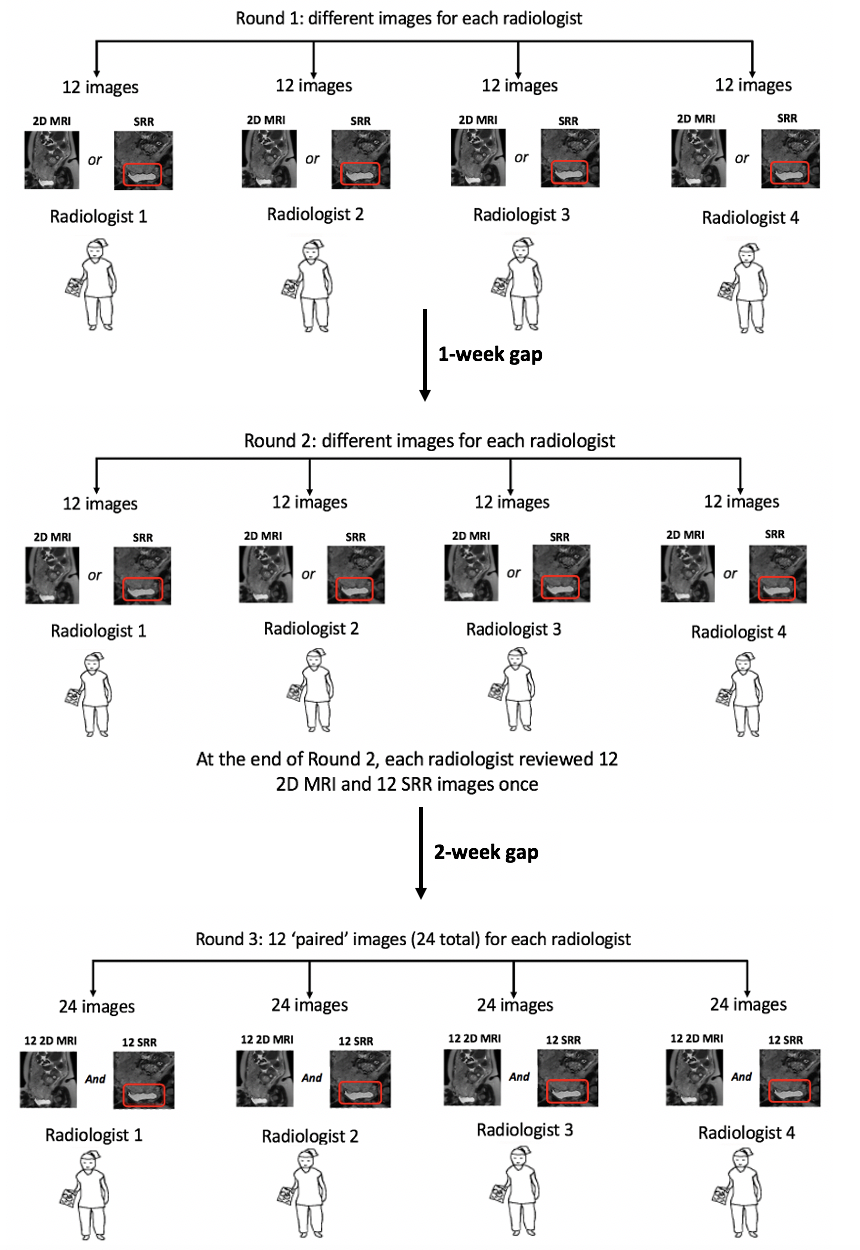


Supplementary Information 1 Flowchart of experimental design. 2D, 2-dimensional; MRI, Magnetic Resonance Imaging; SRR, super-resolution reconstruction.

| **Theme 1: Diagnosis and Confidence of PAS Diagnosis** | |
| --- | --- |
| Question | Scoring System |
| PAS suspected? | 1: Yes  0: No |
| Confidence regarding identifying patients at high risk of PAS diagnosis at birth | 1: not confident at all  2: slightly confident  3: somewhat confident  4: fairly confident  5: completely confident |
| **Theme 2: Anatomical Clarity (Subjective)** | |
| Anatomical Clarity (Placenta-Myometrium-Bladder Interface) | 0: structure not seen  1: poor depiction  2: suboptimal visualisation  3: clear visualisation of structure but reduced tissue contrast; image-based diagnosis feasible  4: excellent depiction; optimal for diagnostic purposes |
| **Theme 3: Identification of Pathological MRI Markers** | |
| T2-dark intraplacental bands | 1: Yes  0: No |
| Placental/uterine buldge | 1: Yes  0: No |
| Loss of retroplacental T2-hypointense line | 1: Yes  0: No |
| Myometrial thinning | 1: Yes  0: No |
| Bladder wall interruption | 1: Yes  0: No |
| Focal exophytic mass | 1: Yes  0: No |
| Abnormal vascularisation of the placental bed | 1: Yes  0: No |
| Placental heterogeneity | 1: Yes  0: No |
| Asymmetric shape/thickening of the placenta | 1: Yes  0: No |
| Placental ischaemic infarction | 1: Yes  0: No |
| Abnormal extraplacental vascularity | 1: Yes  0: No |

Supplementary Information 2 Clinical Questions. PAS, Placenta Accreta Spectrum; MRI, Magnetic Resonance Imaging

| **Subjective Subscale** | **Description** |
| --- | --- |
| Mental Demand | How mentally hurried was the task? |
| Physical Demand | How physically demanding was the task? |
| Temporal Demand | How hurried or rushed was the pace of the task? |
| Performance | How successful were you in accomplishing what you were asked to do? |
| Effort | How hard did you have to work to accomplish your level of performance? |
| Frustration | How insecure, discouraged, irritated, stressed, and annoyed where you? |

Supplementary Information 3 Hart and Staveland’s NASA Task Load Index (TLX) assesses cognitive workload. There is a total of 21 gradations for each subjective subscale. Increments of 7 high, medium, and low estimates result in 21 gradations for each subscale.

Demographics and Surgical Information according to final diagnosis:

|  | **PAS disorder (n=8)** | | **Placenta Praevia (n=4)** | |
| --- | --- | --- | --- | --- |
| **Demographics and Surgical Information** | **Mean ± SD** | **Range** | **Mean ± SD** | **Range** |
| **Age (years)** | 41.25 ± 6.8 | 36-52 | 34 ± 7.07 | 25-42 |
| **Parity** | 2.63 ± 1.06 | 2-9 | 1.75 ± 0.96 | 1-3 |
| **GA at MRI (weeks +days)** | 33^+3^ ± 4^+2^ | 27^+0^ – 38^+6^ | 33^+3^ ± 3^+2^ | 30^+0^ – 36^+5^ |
| **GA at caesarean section (weeks+ days)** | 36^+3^ ± 1^+2^ | 34^+0^ ± 38^+6^ | 36^+0^ ± 1^+1^ | 34^+0^ – 37^+1^ |
| **Number of previous caesarean sections** | 3 ± 1.07 | 1-4 | 1.25 ± 1.26 | 0-3 |
| **Number of previous classical caesarean sections** | 0 | n/a | 0 | n/a |
| **Number of previous surgical uterine evacuations** | 0 | n/a | 1.5 ± 1.91 | 0-4 |
| **Was caesarean scar pregnancy suspected in the 1^st^ Trimester?** | 0.13 ± 0.35 | 0-1 | 0 | n/a |
| **Number of previous Uterine Surgery (e.g., myomectomy, endometrial ablation)** | 0 | n/a | 0 | n/a |
| **Previous history of PAS** | 0 | n/a | 0.25 ± 0.5 | 0-1 |
| **Number of previous scar ectopic** | 0 | n/a | 0 | n/a |
| ***Blood loss at delivery (L)** | 5.01 ± 4.05 | 1.2-14 | 1.5 ± 0.9 | - 1. – 2.5 |

Supplementary Information 4 Demographics and Surgical Information. SD, Standard Deviation; MRI, Magnetic Resonance Imaging; **p*=0.028

| Case | Slice Rejection, mean ± SD (range) | No. of stacks for SRR | SRR quality^†^ |
| --- | --- | --- | --- |
| 1 | 3 ± 3 (0-6) | 5 | 0.75 |
| 2 | 0 | 4 ^‡^ | 0.5 |
| 3 | 22 ± 17 (6-43) | 5 | 0.75 |
| 4 | 9 ± 10 (0-23) | 4 ^‡^ | 0.25 |
| 5 | 4 ± 3 (0-7) | 4 ^‡^ | 0 |
| 6 | 4 ± 4 (1-9) | 4 ^‡^ | 0.5 |
| 7 | 6 ±7 (0-18) | 5 | 0.25 |
| 8 | 6 ± 4 (0-10) | 4 ^‡^ | 0.25 |
| 9 | 1 | 4 ^‡^ | 0.5 |
| 10 | 21 ± 23 (5-61) ^§^ | 6 | 0 |
| 11 | 6 ± 4 (3-10) | 3 ^‡^ | 0.5 |
| 12 | 8 ± 5 (5-14) | 3 ^‡^ | 0 |

Supplementary Information 5

SRR, super-resolution reconstruction; SD, Standard Deviation

† Average obtained across 4 radiologists whereby 0 = lots of artifact/blur, 1 = little artifact/blur, and 2 = no artifact/blur.

‡ Fewer than 5 stacks are typically insufficient for obtaining good SRR quality.

§ A large number of slice rejection despite a sufficient number of stacks leading to suboptimal SRR quality


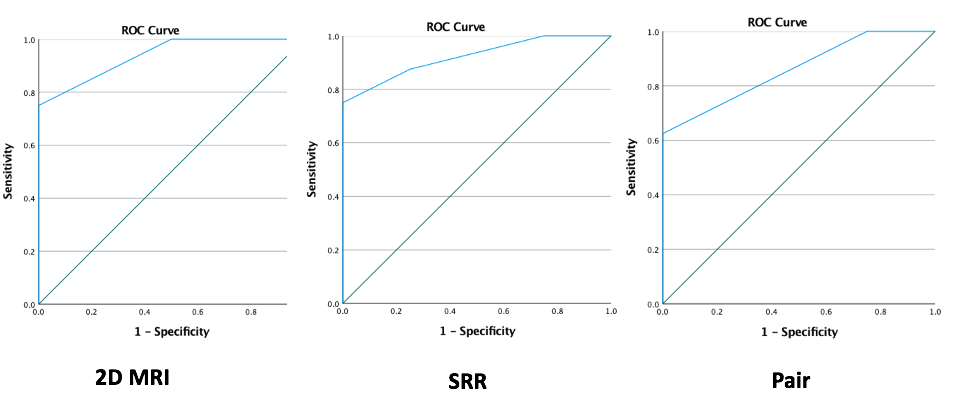


Supplementary Information 6 ROC Curve analysis of a high probability of PAS at birth is displayed for all imaging modalities.

2D, 2-dimensional; MRI, Magnetic Resonance Imaging; SRR, Super Resolution Reconstruction.


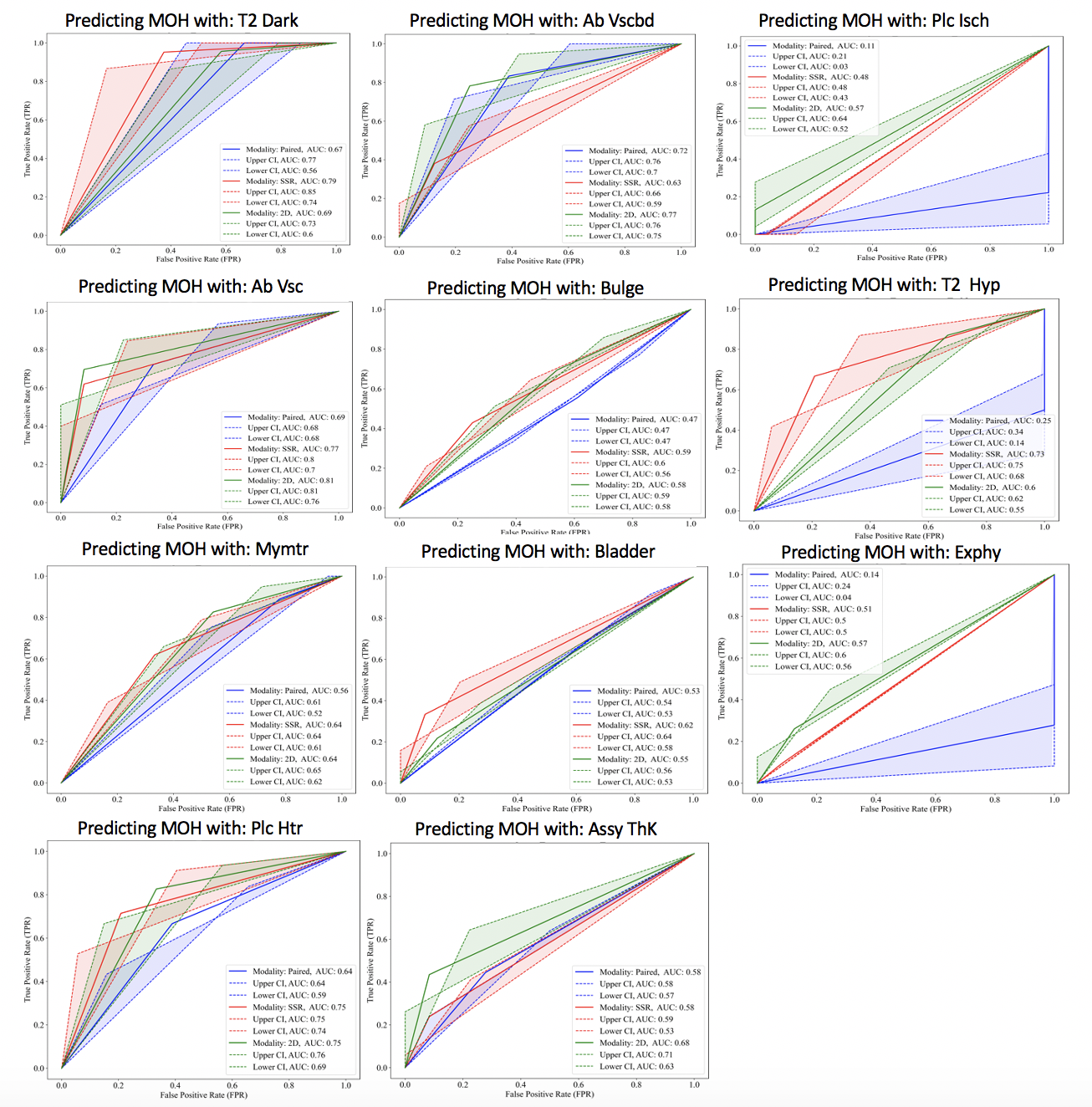


Supplementary Information 7 Major Obstetric Haemorrhage (MOH) predictivity displayed using ROC Curve Analysis for each abnormality of utero placenta circulation, and pathological MRI marker of placenta accreta spectrum are displayed using each imaging modality: 2D MRI (2D) (green), Super-Resolution Reconstruction (SRR) (red), and Paired Imaging (blue). It is expected that the AUC would be less when using an individual MRI marker as opposed to the most predictive (lowest ranking) MRI markers collectively.

T2 Dark, T2-dark intraplacental bands; Ab Vscbd, Abnormal vascularisation of the placental bed; Plc Isch, Placental ischaemic infarction; Ab Vsc, Abnormal extraplacental vascularity; Bulge, Placental/uterine buldge; T2 Hyp, Loss of retroplacental T2-hypointense line; Mymtr, Myometrial thinning; Bladder, Bladder wall interruption; Exphy, Focal exophytic mass; Plc Htr, Placental heterogeneity; Assy ThK, Asymmetric shape/thickening of the placenta.


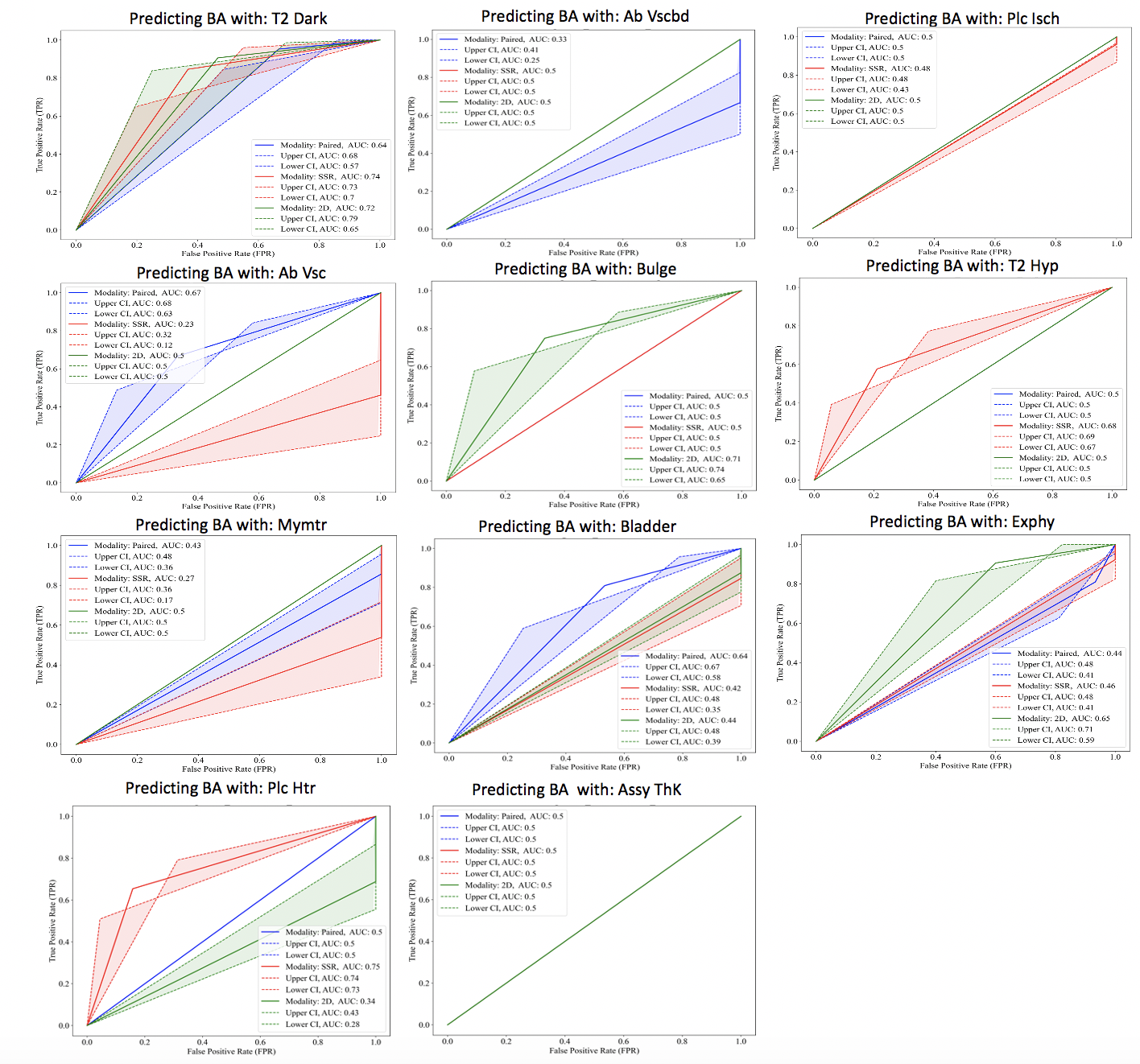


Supplementary Information 8 Moderate/severe bladder adherence (BA) predictivity displayed using ROC Curve Analysis for each abnormality of utero placenta circulation, and pathological MRI marker of placenta accreta spectrum are displayed using each imaging modality: 2D MRI (2D) (green), Super-Resolution Reconstruction (SRR) (red), and Paired Imaging (blue). It is expected that the AUC would be less when using an individual MRI marker as opposed to the most predictive (lowest ranking) MRI markers collectively.

T2 Dark, T2-dark intraplacental bands; Ab Vscbd, Abnormal vascularisation of the placental bed; Plc Isch, Placental ischaemic infarction; Ab Vsc, Abnormal extraplacental vascularity; Bulge, Placental/uterine buldge; T2 Hyp, Loss of retroplacental T2-hypointense line; Mymtr, Myometrial thinning; Bladder, Bladder wall interruption; Exphy, Focal exophytic mass; Plc Htr, Placental heterogeneity; Assy ThK, Asymmetric shape/thickening of the placenta.


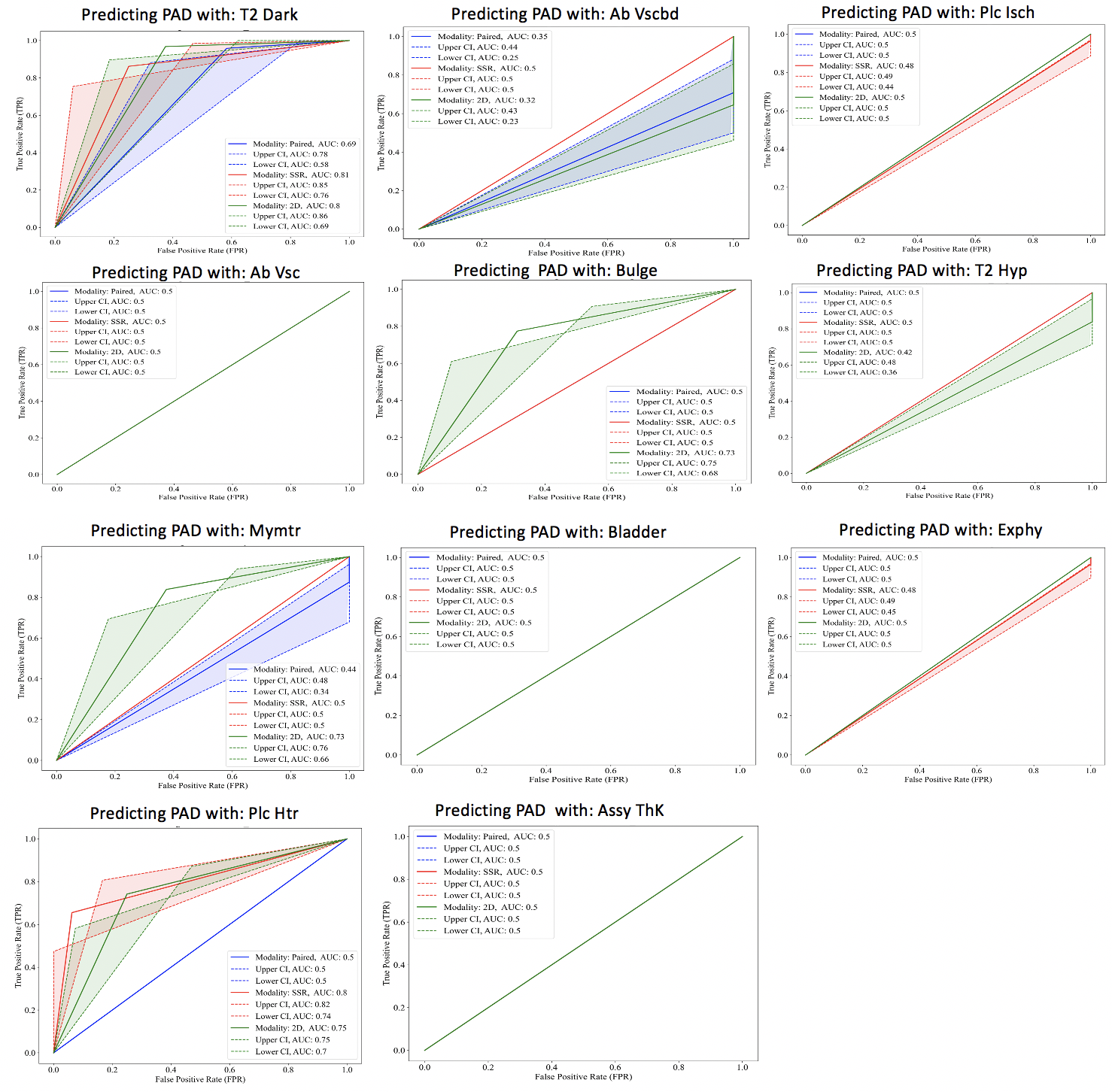


Supplementary Information 9 Superficial/deep placental attachment depth (PAD) predictivity displayed using ROC Curve Analysis for each abnormality of utero placenta circulation, and pathological MRI marker of placenta accreta spectrum are displayed using each imaging modality: 2D MRI (2D) (green), Super-Resolution Reconstruction (SRR) (orange), and Paired Imaging (blue). It is expected that the AUC would be less when using an individual MRI marker as opposed to the most predictive (lowest ranking) MRI markers collectively.

T2 Dark, T2-dark intraplacental bands; Ab Vscbd, Abnormal vascularisation of the placental bed; Plc Isch, Placental ischaemic infarction; Ab Vsc, Abnormal extraplacental vascularity; Bulge, Placental/uterine buldge; T2 Hyp, Loss of retroplacental T2-hypointense line; Mymtr, Myometrial thinning; Bladder, Bladder wall interruption; Exphy, Focal exophytic mass; Plc Htr, Placental heterogeneity; Assy ThK, Asymmetric shape/thickening of the placenta.


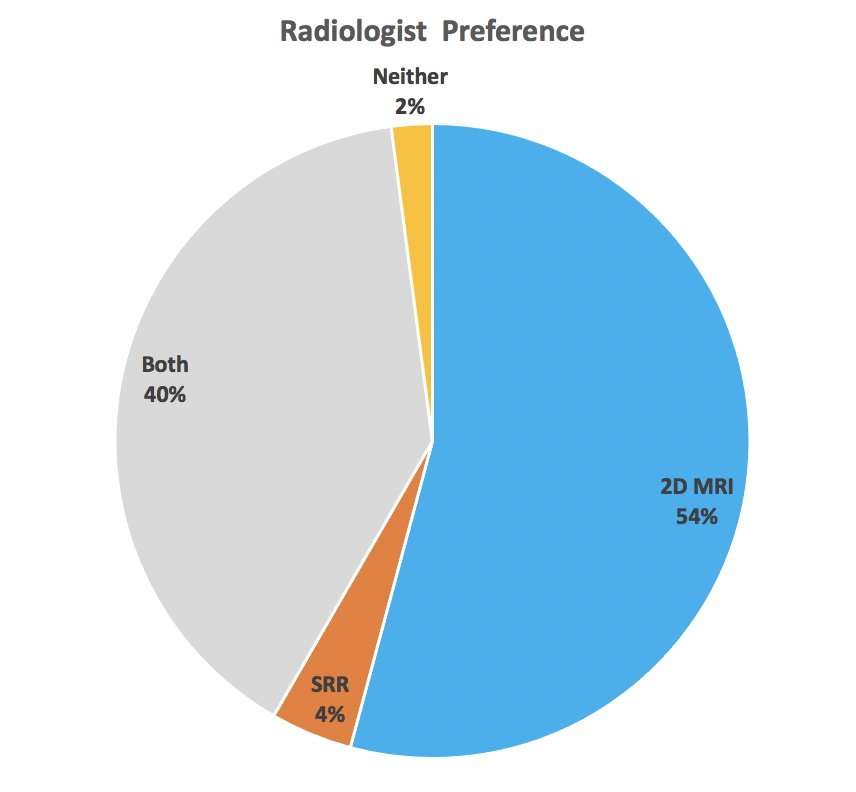


Supplementary Information 10 Pie chart to indicate radiologist preference in using 2D MRI alone, SRR alone, both, and neither.

Supplementary information 11

This is an animation of a 3-D MRI model of a case with placenta accreta spectrum. The purple mesh represents the myometrium, the magenta mesh represents placental vasculature, and the green mesh represents the placenta.
